# Antibacterial, antioxidant, antifungal and anti-inflammatory activities of crude extract from *Nitraria schoberi* fruits

**DOI:** 10.1007/s13205-014-0266-1

**Published:** 2014-11-13

**Authors:** Javad Sharifi-Rad, Seyedeh Mahsan Hoseini-Alfatemi, Majid Sharifi-Rad, Jaime A. Teixeira da Silva

**Affiliations:** 1 Zabol Medicinal Plants Research Center, Zabol University of Medical Sciences, Zabol, Iran; 2 Department of Pharmacognosy, Faculty of Pharmacy, Zabol University of Medical Sciences, Zabol, Iran; 3 Pediatric Infections Research Center, Mofid Children Hospital, Shahid Beheshti University of Medical Sciences, Tehran, Iran; 4 Department of Range and Watershed Management, Faculty of Natural Resources, University of Zabol, Zabol, Iran; 5 Miki-cho Post Office Retired, Ikenobe 3011-2, P.O. Box 7, Kagawa-ken, 761-0799 Japan

**Keywords:** Agar disc diffusion method, Antifungal activity, Anti-inflammatory activity, Antioxidant activity, Antimicrobial activity, Zygophyllaceae

## Abstract

This study is the first comprehensive investigation of the antibacterial, antioxidant, antifungal and anti-inflammatory activities of a crude extract from *Nitraria schoberi* L. (Zygophyllaceae) fruits. The extract was tested against four Gram-negative (*Pseudomonas aeruginosa*, *Enterobacter aerogenes*, *Klebsiella pneumoniae* and *Acinetobacter lwoffii*) and one Gram-positive (*Staphylococcus aureus*) bacteria using the agar disc diffusion and microdilution methods. *P. aeruginosa* was inhibited the most (widest inhibition zone) while *K. pneumonia* showed the largest MIC value. The antioxidant activity of fruits (0.02 mg/mL vs. 0.04, 0.06 and 1.00 mg/mL for α-tocopherol, butylated hydroxyanisole and ascorbic acid, respectively) was determined by the paired diene method. The antifungal activity of *N. schoberi* fruits was tested against different fungi, including *Aspergillus niger* and *Candida albicans*, with 300 µg/mL of fruit extract being the most effective concentration. The percentage of anti-inflammatory activity assayed for *N. schoberi* fruit extract at 100, 200 and 500 µg/mL was 36.12, 59.89 and 88.33 %, respectively. *N. schoberi* fruits possess potent antibacterial, antioxidant, antifungal and anti-inflammatory properties, and may be used as an antibacterial and antifungal to treat diseases and/or as a protective agent against disorders associated with oxidative stress and inflammation.

## Introduction

Plants play a significant role in supplying food for humans. Medicinal and aromatic plants have played a critical role as therapeutic agents for a long time and thus hold great economic value (Balunas and Kinghorn 2005). There is a rising tendency to use natural drugs and herbal therapies, partly because of the destructive nature of and side effects of chemical drugs, as well as environmental pollution (Balunas and Kinghorn 2005; Rad et al. 2013a; Sharifi-Rad et al. 2014). Many naturally occurring agents in plant extracts show antimicrobial, antioxidant, antifungal, anticancer and anti-inflammatory potential in several animal models and bioassay systems, and are pertinent to human disease (Patwardhan 2005; Rad et al. 2014a).

*Nitraria schoberi* L. (Zygophyllaceae) is a medical plant. *Nitraria* plants, specifically the leaves, fruits and seeds, are often used in folklore medicine as an antispasmodic, and contain several classes of secondary metabolites including sterols, fatty acids, alkaloids and flavonoids derivatives (Suo and Wang 2010; Hadj et al. 2011). The aims of this study were to investigate the antimicrobial activity of a fruit extract against four Gram-negative and one Gram-positive bacteria as well as its antifungal and anti-inflammatory activities.

## Materials and methods

### Preparation of *Nitraria schoberi* fruit extracts

**Fig. 1:**
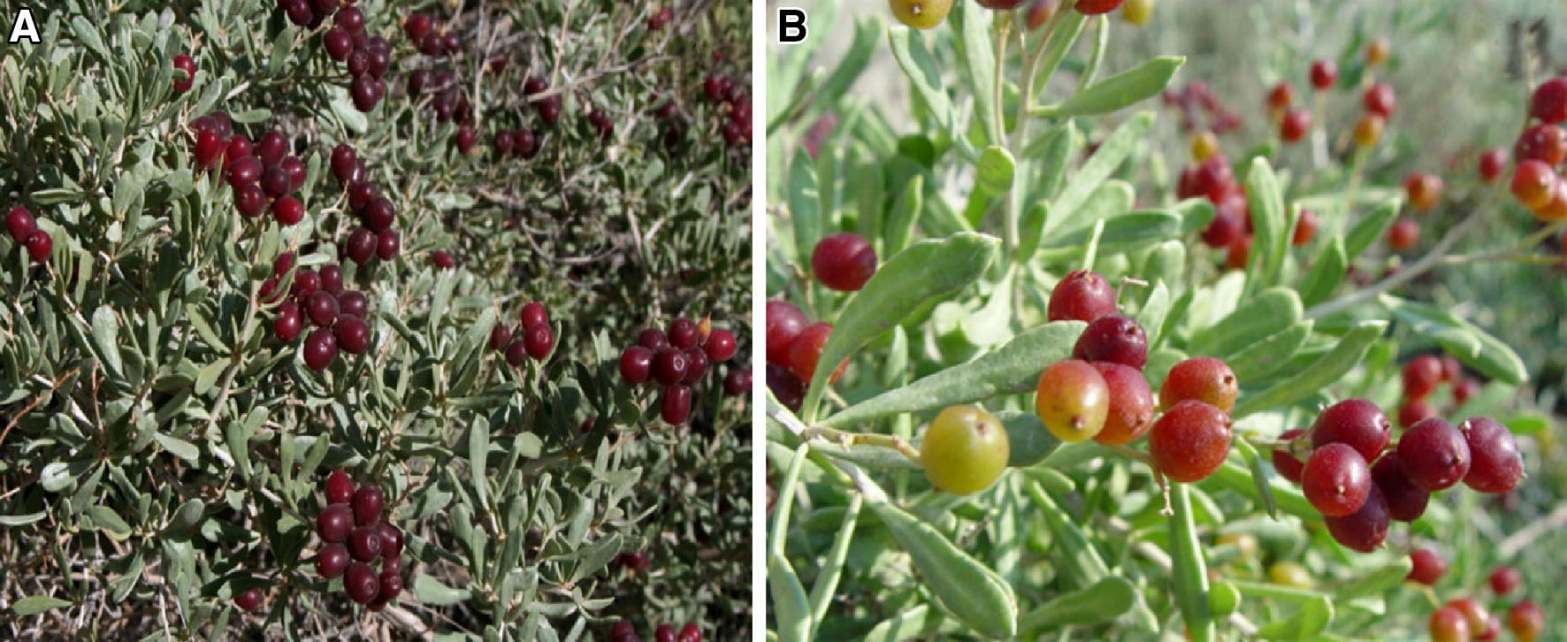
*Nitraria schoberi* plant used for extract analyses in this study. **a** Wild population; **b** Close-up of plant

Fresh fruits of *N. schoberi* plants (Fig. 1a, b) were collected from Gonabad (coordinates: 34°21′10″N, 58°41′01″E), Razavi Khorasan Province, Iran in June 2013. The plant was taxonomically identified by a botanist at the herbarium of Pharmacognosy, Department of the Faculty of Pharmacy affiliated to Shahid Beheshti University of Medical Sciences of Iran. All fruits were thoroughly washed in distilled water three times then dried in an oven at 75 °C for 48 h. The dried fruits were powdered by a mechanical grinder and then 20 g of powdered was dissolved in 200 mL of 85 % methanol using a shaking water bath (50 rpm) for 24 h at room temperature. After filtering through Whatman No. 1 filter paper, the filtrate was concentrated with a rotary evaporator (Laborota 4000, Heidolph, Germany) at 45 °C for 35 min to remove solvent from the extract. The solid-like material that precipitated (i.e., the extract) was stored at 4 °C until further analysis.

### Microorganisms

The microorganisms used in this study were obtained from the microbiological laboratory of MRI Hospital in Shiraz (Iran). These bacteria were isolated from MRI patients that provided written permission to do so. Four Gram-negative (*Pseudomonas aeruginosa*, *Enterobacter aerogenes*, *Klebsiella pneumoniae* and *Acinetobacter lwoffii*) and one Gram-positive (*Staphylococcus aureus*) bacteria were inoculated onto nutrient agar slants at 37 °C and maintained at −80 °C.

### Antibiotic discs and microdilution assay

Antimicrobial activity was based on the disc diffusion method (Thitilertdecha et al. 2008) utilizing a separate cell suspension of each microorganism. The concentration of each cell suspension was equilibrated to a 0.5 McFarland standard and 50 μL of each microorganism’s suspension was spread on a Mueller–Hinton agar plate. In addition, 50 μL of diluted fruit extract (1 g/5 mL of distilled water) was pipetted onto sterile blank discs (6 mm in diameter), which were allowed to dry in an open sterile Petri dish in a biological laminar flow bench. Discs were placed on the surface of inoculated plates and incubated at 37 °C for 24 h. Diameters (mm) of the zones of bacterial inhibition minus the discs diameter were recorded (Aureli et al. 1992). Cephalosporin discs (Padtan Teb Co., Iran) were used as positive controls. Cephalosporin is an antibiotic with antibacterial activity that inhibits cell wall synthesis although some bacteria such as *Klebsiella* spp., *Proteus* spp. and *Pseudomonas* sp. are resistance to it (Wright 2010).

The minimal inhibitory concentration (MIC) values of the plant extract versus each investigated microbial strain were determined by the microdilution assay in 96 multi-well microtiter plates according to the standard procedure of the Clinical and Laboratory Standards Institute (CLSI 2010). All assays were carried out in Mueller–Hinton Broth (MHB). The plant extract were dissolved in 5 % dimethyl sulfoxide (DMSO) to a final concentration of 10 mg/mL. Each strain was assayed with samples that were serially diluted in broth to obtain concentrations ranging from 512.0, 256, 128, 64, 32, 16, 8, 4, 2, 1, 0.5, 0.25, 0.12 and 0.06 µg/mL. Overnight broth cultures of each strain were prepared and the final microorganism concentration in each well was adapted to 10^6^ CFU/mL. The optimal incubation conditions were 37 °C for 24 h. The antimicrobial activity to the plant extracts was classified according to the MIC values as follows: MIC >1,000 µg/mL, no antimicrobial activity; 512 ≤ MIC ≤ 1,000 µg/mL, mild activity; 128 ≤ MIC < 512 µg/mL, moderate activity; 32 ≤ MIC < 128 µg/mL, good activity; 10 ≤ MIC < 32 µg/mL, strong activity; and MIC <10 µg/mL, very strong activity (CLSI 2010). The experiment was carried out in duplicate and the results were expressed as average values.

### Antioxidant activity of fruits

The antioxidant activity of fruits was determined by the paired diene method (Lingnert et al. 1979). The antioxidant activity measured is the capacity of the extract to inhibit the peroxidation of linoleic acid in which the double bond is altered to a paired diene. Each extract sample (0.01–30 mg/mL) in methanol (100 µL) was mixed with 3 mL of 10 mM linoleic acid (Sigma Chemical Co., St. Louis, MO, USA) to form an emulsion in 0.2 M sodium phosphate buffer (pH 6.6) in test tubes and placed in the dark at 37 °C to hasten oxidation. After incubation for 15 h, 7 mL of 65 % methanol in deionized water was added, and the absorbance of the mixture was measured at 234 nm against a blank in a Hitachi U-2001 spectrophotometer (Tokyo, Japan). Antioxidant activity was quantified as follows: antioxidant activity (%) = [(∆*A*_234_ of control – ∆*A*_234_ of sample)/∆*A*_234_ of control] × 100. Analyses were repeated three times. α-Tocopherol, butylated hydroxyanisole (BHA) and ascorbic acid (Sigma) were used as standard controls. Antioxidant activity was expressed as the EC_50_ value (mg/mL), which is the effective concentration at which the antioxidant activity was inhibited by 50 %, gained by interpolation from linear regression analysis.

### Antifungal activity

Antifungal activity of *N. schoberi* fruits extracts was assayed against two fungi (*Aspergillus niger* ATCC 9142 and *Candida albicans* ATCC 10231). The fungi were cultured at 37 °C for 14–24 h and the densities were adjusted to 0.5 McFarland standards at A_530_ nm (10^8^ colony-forming units (CFU)/mL). The antifungal assays were carried out by the disc diffusion method (Bauer et al. 1996). 100 µL of the microbial suspensions (10^8^ CFU/mL) was spread on nutrient agar (Merck, Germany) plates (100 mm × 15 mm). Discs (6 mm diameter) were impregnated with 10 µL of different concentrations of extract (50, 100, 150, 200, 250 and 300 µg/mL) and placed on the inoculated agar. All the inoculated plates were incubated at 37 °C for 24 h. Positive control discs comprised ketoconazole (50 mcg/disc) (Padtan Teb Co., Iran) for fungi. In addition, two microliters of 5 % (v/v) DMSO was used as the negative control. Antifungal activity was determined by measuring the zone of inhibition (in mm).

MIC was determined using serial dilutions of the extract (512–0.05 µg/mL) using the microdilution test confirmed by Clinical and Laboratory Standards Institute (Wayne 2006). The fungal strains were suspended in Luria–Bertani medium and the densities were regulated to 0.5 McFarland standards at 570 nm (10^8^ CFU/mL). Fungal suspensions (100 µL) and extracts were added to microtiter plates and incubated at 37 °C for 24 h. The sterile control was medium without fungi while the growth control was medium with fungi, but without extract. The growth in each well was compared with that in the control well. MICs were visually detected in comparison with growth of the control well and delineated as the lowest concentration of the extract that inhibited growth.

### Anti-inflammatory activity

The anti-inflammatory activity of *N. schoberi* fruits was tested by the protein denaturation method as described by Padmanabhan and Jangle (2012). Briefly, the reaction included 1 mL of various concentrations of *N. schoberi* fruits extracts (100, 200 and 500 µg/mL in distilled water) and 3 mL of phosphate-buffered saline (pH 6.5) which were blended with 2 mL of egg albumin and incubated at 25 °C for 15 min. A denaturation reaction was induced in a 65 °C water bath for 12 min. After cooling, absorbance was measured at 660 nm (*A*_660_) with a Shimadzu A160 spectrofluorometer (Shimadzu, Japan) using double distilled water as the blank. The percentage inhibition of protein denaturation was appraised by the following formula (Padmanabhan and Jangle 2012):

$$\%\,\text{inhibition}=[A_{\rm s}-A_{\rm c}/A_{\rm c}] \times 100$$

where *A*_s_ and *A*_c_ represent sample and control absorbance, respectively.

In this assay, diclofenac sodium (Padtan Teb Co., Iran), a powerful non-steroidal anti-inflammatory drug, was used as the standard drug in the same concentrations as *N. schoberi* fruit methanol extracts (100, 200 and 500 µg/mL in distilled water).

### Statistical analysis

The extract was prepared in triplicate for antibacterial, antioxidant, antifungal and anti-inflammatory activities tests. Data were subjected to analysis of variance following a completely random design to determine the least significant difference (LSD) at *P* < 0.05 using SPSS v. 11.5 (IBM SPSS, New York, USA).

## Results and discussion

### Antibacterial activity

**Fig. 2:**
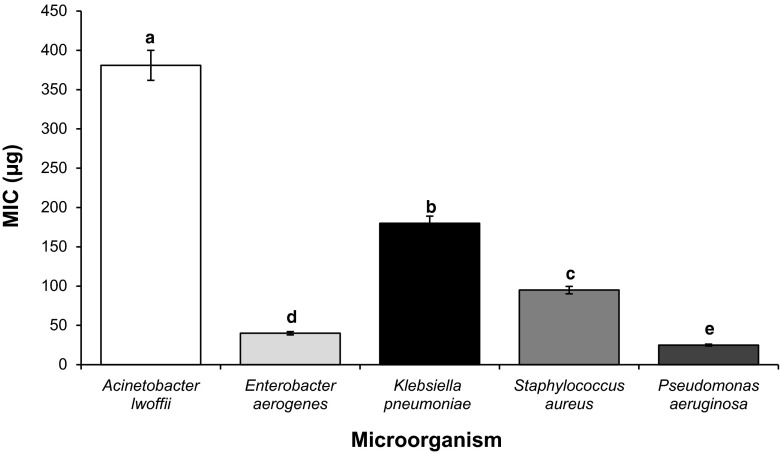
MIC values of *Nitraria schoberi* methanolic fruit extract against five tested bacteria. Values are mean of ±SE of three replicates. Means with *different letters* within a column are significantly different (*P* < 0.05; LSD)

The fruits of *N. schoberi* exhibit varying degrees of inhibition against all bacteria tested (Table 1). Inhibition zones of *A. lwoffii*, *E. aerogenes*, *K. pneumonia*, *S. aureus* and *P. aeruginosa* were 7, 18, 14, 16 and 25 mm, respectively, indicating that *N. schoberi* fruit extract has a maximum effect on *P. aeruginosa* and a minimum effect on *A. lwoffii. P. aeruginosa* and *A. lwoffii* were resistant to cephalosporin (control drug), but sensitive to *N. schoberi* fruit extract (Table 1). The MIC of *A. lwoffii*, *E. aerogenes*, *K. pneumonia*, *S. aureus* and *P. aeruginosa* was 138, 40, 180, 95 and 25 µg/mL, respectively. This result confirmed the disc diffusion data since minimum MIC and maximum MIC were related to *P. aeruginosa* and *A. lwoffii*, respectively (Fig. 2).

**Table 1:** Mean diameters of inhibition zone with *Nitraria schoberi* methanol fruit extract and two positive controls

Microorganism	Inhibition zone diameter (mm)	
	Fruit extract	Cephalosporin
*Acinetobacter lwoffii*	7 ± 0.05	0 ± 0.00
*Enterobacter aerogenes*	18 ± 0.17	5 ± 0.57
*Klebsiella pneumoniae*	14 ± 0.08	0 ± 0.00
*Staphylococcus aureus*	16 ± 1	4 ± 0.21
*Pseudomonas aeruginosa*	25 ± 0.57	0 ± 0.00

Values are mean of ± SE of three replicates

Bacteria that are resistant to antibiotics may jeopardize medicine. Some such antibiotic-resistant bacteria have greater importance than others, including *S. aureus*, *A. lwoffii*, *E. aerogenes*, *K. pneumonia* and *P. aeruginosa* (Chopra 2007). *S. aureus*, a Gram-positive bacterium, is a highly changeable pathogen in response to antibiotics with considerable importance in human medicine (Alfatemi et al. 2014). It is responsible for a wide range of hospital and community-acquired infections globally, ranging from skin infections and food poisoning to life-threatening situations such as toxic-shock syndrome, endocarditis, pneumonia, bacteremia and osteomyelitis (Kim et al. 2006; Pottinger 2013; Ray et al. 2013; Zecconi and Scali 2013). *A. lwoffii* is widespread, and a comparatively harmless organism with the ability to persist in a hospital environment for prolonged periods (Rathinavelu et al. 2003; Garn et al. 2013). *Enterobacter aerogenes* causes bacterial infections, and is usually acquired in a hospital or in hospital-type atmospheres. *Enterobacter aerogenes* usually brings about opportunistic infections, implying that it will usually only cause an illness in a person or host that has a compromised immune system although studies are now showing that it is causing increased alarm in community infections even though it seldomly causes disease in a person with a healthy immune system (Lavigne et al. 2013). *Klebsiella pneumonia*, responsible for nosocomial pneumonia in “immunoincompetent” people, produces approximately 10 % of all infections that are acquired in hospitals including urinary tract infections, pneumonia, surgical wounds, and biliary tract wounds (Tam 2013). The most important Gram-negative bacterium is *P. aeruginosa*, which has become a significant cause of infection, particularly in patients with compromised host defense mechanisms (Tepe et al. 2004; Jeyaraj et al. 2013). *Pseudomonas aeruginosa* is the main and usual pathogen isolated from patients who have been hospitalized for longer than 1 week (Ahmed et al. 2013). It is a frequent cause of nosocomial infections such as pneumonia, urinary tract infections, and bacteremia. Pseudomonal infections are complicated and can be life threatening. Gram-positive bacteria such as *S. aureus* are primarily responsible for post-operative wound infections, toxic-shock syndrome, endocarditis, osteomyelitis and food poisoning (O’Malley et al. 2009).

Studies on the discovery of natural antibacterial sources from plants are increasing. Ahmad and Beg (2001) studied the ethanolic extracts of 45 Indian medicinal plants customarily used for their antimicrobial activity against specific drug-resistant bacteria and a yeast *C. albicans* of clinical origin. 40 of these plant extracts showed different levels of antimicrobial activity against one or more of the assayed bacteria, anticandidal activity was observed in 24 plant extracts while broad-spectrum antimicrobial activity was discovered in 11 plants (*Lawsonia inermis*, *Eucalyptus* sp., *Holarrhena antidysenterica*, *Hemidesmus indicus*, *Casuarina equistifolia*, *Terminalia belerica*, *T. chebula*, *Emblica officinalis*, *Camelia sinensis*, *Syzgium* *aromaticum*, and *Punica granatum*). No similarity was observed between the vulnerability of test strains with plant extracts and antibiotic resistance of the microbial strains (*Salmonella paratyphi*, *Shigella dysenteriae*, *S. aureus*, *Bacillus subtilis*, *Escherichia coli*, *C. albicans*). Qualitative phytochemical examinations, thin layer chromatography (TLC) and a TLC-bioautography of specific active extracts exhibited the presence of ordinary phytocompounds in the plant extracts, including phenols, tannins and flavonoids as the major active components.

Mabona et al. (2013) investigated the antimicrobial characteristics of southern African medicinal plants against dermatologically relevant pathogens. Plants exhibiting noteworthy broad-spectrum activities (MIC values ≤1.00 mg/mL) against the assayed pathogens comprised extracts from *Diospyros mespiliformis*, *Aristea ecklonii*, *Chenopodium ambrosioides*, *Eucalyptus camaldulensis*, *Elephantorrhiza elephantina*, *Gunnera perpensa*, *Harpephyllum caffrum*, *Melianthus comosus*, *Hypericum perforatum*, *Warburgia salutaris* and *Terminalia sericea*. The organic extract of *E. elephantina*, a plant reportedly used to treat common acne (*Propionibacterium acnes*), demonstrated considerable antimicrobial activity (MIC = 0.05 mg/mL) against *P. acnes*. Similarly, *D. mespiliformis*, traditionally used to treat ringworm, also displayed antimicrobial activity against *Trichophyton mentagrophytes* (MIC = 0.10 mg/mL) and *Microsporum canis* (MIC = 0.50 mg/mL). The aqueous root extracts of *P. prunelloides* combined with *E. elephantina* in a 1:1 (v/v) ratio showed synergistic interactions against *S. aureus*, gentamycin–methicillin resistant *S. aureus*, *Staphylococcus epidermidis* and *C. albicans*. Fractionation of *A. ecklonii* resulted in the separation of plumbagin, exhibiting remarkable antimicrobial activity in which the MIC ranged from 2.00 to 16.00 μg/mL. Rad et al. (2013b) evaluated the antibacterial activity of stem and flower essential oils from *Sinapis arvensis* on five pathogenic multidrug-resistant bacteria strains by the agar disc diffusion method. The Gram-positive and Gram-negative bacteria that were most susceptible to the stem and flower essential oils of *S. arvensis* were *S. aureus* (NCTC7428) and *P. aeruginosa* (MTCC 2453), respectively.

Previous studies have shown that antibacterial activity is caused by various classes of secondary metabolites, including fatty acids, sterols, alkaloids and flavonoids derivatives, flavonoids and phenolic compounds (Tulyaganov and Allaberdiev 2003; Suo and Wang 2010; Hadj et al. 2011). In earlier studies, it was determined that *N.* *sibirica* fruits contained phenolic compounds (Senejoux et al. 2012).

### Antioxidant activity

The results for antioxidant activity of the extract tested are summarized in Table 2. The efficiency of antioxidant activity is inversely related with the extract’s EC_50_ values. The antioxidant activity, i.e., EC_50_ values, was 0.04, 0.06, 1.00 and 0.02 mg/mL for α-tocopherol, butylated hydroxyanisole (BHA), ascorbic acid and *N. schoberi* fruits, respectively (Table 2). The fruit extract was not significantly different to α-tocopherol and BHA (*P* < 0.05), but only significantly different to ascorbic acid (*P* < 0.05).

**Table 2:** EC_50_ values (mg/mL) of the *Nitraria schoberi* methanolic fruit extract in two assays

	Antioxidant activity
Fruit extract	0.02 ± 0.00 b
α-Tocopherol	0.04 ± 0.00 b
BHA	0.06 ± 0.02 b
Ascorbic acid	1.00 ± 0.00 a

Values are mean of ±SE of three replicates. Means with different letters within a column are significantly different (*P* < 0.05; LSD)

Rad et al. (2014b) investigated the free radical-scavenging and antioxidant activities of different parts of *N. schoberi* plants collected from Zabol, Sistan and Baluchestan Province, Iran. They showed that the maximum free radical and antioxidant activities were associated with the methanolic extracts rather than with other extracts (aqueous and chloroform). These activities were maximum in fruits, and lower in leaves and roots. The methanolic, aqueous and chloroform extracts showed 86, 70 and 57 % scavenging activity, respectively. In this study, we investigated the fruits of *N. schoberi* collected from Gonabad, Razavi Khorasan Province, Iran, and also observed antioxidant activity, indicating that this activity remains constant, independent of the geographic location. *N. schoberi* may be considered a broad and effective source of healthy antioxidants and bioactive phytochemicals.

Choi et al. (2002) evaluated the root bark and leaves of several Korean medicinal plants to appraise their free radical-scavenging capacity and antioxidant activity using common assays with dichloromethane, ethanol or methanol extracts. Flavonoids, including catechin, morin, naringenin, quercetin and rutin, were included and used as standards. Among the plant extracts, the root bark of *Morus alba* L. and the leaves of *Saururus chinensis* showed stronger values than other plant extracts.

Bouayed et al. (2007) conducted a comparative study between the leaves of *Lavandula officinalis* L., the roots of *Verbena officinalis* L., the flowers of *Calylophus lavandulifolius*, the leaves of *Melissa officinalis* L. and the flowers of *Althea kurdica* plants from the same geographic origin, the Hamadan region in the west of Iran and growing in the same natural conditions. The antioxidant activities differed significantly between all plant parts while some plants were rich in natural antioxidants, especially the leaves of *L. officinalis* and *M. officinalis*.

Nivas et al. (2010) investigated the methanolic extract of young and mature leaves of nine coastal plants from the West coast of Maharashtra, India to appraise their free radical-scavenging activity. The highest antioxidant activity was recorded in young leaves of *Hibiscus tiliaceus* L. (76 %), followed by *Syzigium corymbosa* (71 %), *Calophyllum inophyllum* L. (68 %) and *Colubrina asiatica* (L.) Brongn. (55 %). The leaves of all these species were rich in flavonoids (6.03–16.63 mg/g of dry weight) and total polyphenols (12.12–26.23 mg/g of dry weight). These compounds mainly contributed to the antioxidant potential of these plants.

The above evidence from the literature suggests that some plants have potent antioxidant activity and free radical-scavenging properties. Plant phenolic compounds have powerful antioxidant activity and may assist in the protection of cells against oxidative damage caused by free radicals, which, with other reactive oxygen species, are a causative factor of different diseases such as asthma, mongolism, arthritis, carcinoma, dementia and Parkinson’s disease (Perry et al. 2000).

### Antifungal activity

The antifungal activity of *N. schoberi* fruit extract against two fungi strains is shown in Table 3. Inhibition zones of *A. niger* in response to 50, 100, 150, 200, 250 and 300 µg/mL of fruit extract were 2.1, 5.6, 9.2, 14.5, 15.7 and 18.2 mm, respectively. Inhibition zones of *C. albicans* in response to 50, 100, 150, 200, 250 and 300 µg/mL of fruit extract were 1, 2.5, 6.3, 13.8, 15.1 and 17.9 mm, respectively. DMSO (negative control) and ketoconazole (positive control) showed inhibition zones of 1.0 mm and 14.7 mm, respectively, for *A. niger* or 0.5 mm and 21.1 mm, respectively, for *C. albicans*. The MIC of *A. niger* and *C. albicans* was 284 µg/mL and 342 µg/mL, respectively.

**Table 3:** Antifungal activity of *Nitraria schoberi* fruit extracts against two fungal strains

Fruit extract (µg/mL)	*Aspergillus niger*	*Candida albicans*
50	2.1 ± 0.0	1.0 ± 0.0
100	5.6 ± 0.0	2.5 ± 0.2
150	9.2 ± 0.1	6.3 ± 0.0
200	14.5 ± 0.0	13.8 ± 0.0
250	15.7 ± 0.0	15.1 ± 0.0
300	18.2 ± 0.7	17.9 ± 0.0
DMSO (negative control)	1.0 ± 0.0	0.5 ± 0.0
Ketoconazole (positive control)	14.7 ± 0.0	21.1 ± 0.0
MIC	284.5 ± 0.3	342 ± 0.0

Data are expressed as mean ± SE of inhibition zone diameter (mm) for different concentration of extract, controls and minimum inhibitory concentration (MIC) (µg/mL)

*DMSO* Dimethyl sulfoxide

In the past decade, the prevalence of resistance to antifungal agents has increased. Resistance to antifungal agents has important implications for healthcare, morbidity and mortality in human life. Moreover, a search for new, more secure and potent agents to combat critical fungal infections is required. Plants, especially medicinal plants, are as a good candidate for this aim. In recent years, several studies have been reported on the antifungal activity of phenols, flavonoids, coumarins, quinones, saponins, xanthones, alkaloids, lectins, polypeptides, terpenoids and essential oils from natural sources (Arif et al. 2009). Previous studies showed the presence of secondary metabolites, including alkaloids and flavonoids derivatives, in the Zygophyllaceae (Tulyaganov and Allaberdiev 2003; Hadj et al. 2011).

### Anti-inflammatory activity

**Fig. 3:**
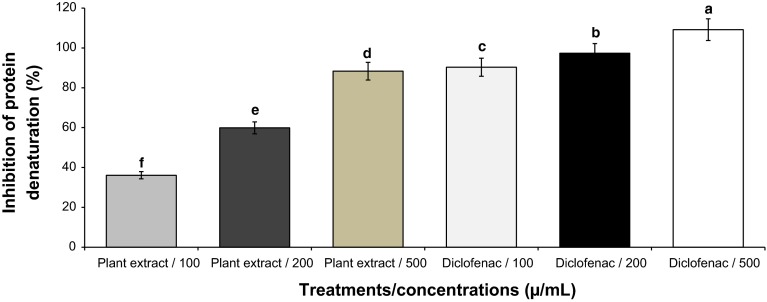
Anti-inflammatory effect of *Nitraria schoberi* fruit extract. Values are mean ± SE of three replicates; means with *different letters* within a column are significantly different (*P* < 0.05; LSD)

The results of anti-inflammatory assay of *N. schoberi* fruit extract are shown in Fig. 3: 36.12, 59.89 and 88.33 %, respectively, for 100, 200 and 500 µg/mL of *N. schoberi* fruit extract. The anti-inflammatory percentage of diclofenac (control) at 100, 200 and 500 µg/mL was 90.31, 97.33 and 109.13 %, respectively. The percentage of inhibition of protein denaturation by *N. schoberi* fruit extracts was significantly different in all treatments (*P* < 0.05).

Inflammation is clinically defined as a pathophysiological procedure described by redness, fever, edema, loss of function and pain (Hyun et al. 2004). Flavonoids possess several biological and pharmacological activities: antimicrobial, anti-inflammatory, immunomodulatory, antiviral, anticancer and antithrombotic activities (Havsteen 1983). Many studies verified that flavonoids possess anti-inflammatory activity in several inflammation animal models. Flavonoids can regulate cellular activities of inflammation-related cells such as macrophages, lymphocytes, neutrophils and mast cells. For example, some flavonoids inhibit T cell proliferation while others inhibit the release of histamine from mast cells (Hyun et al. 2004).

## Conclusion

*Nitraria schoberi* fruits show promising use as new pharmaceuticals with antibacterial, antioxidant, antifungal and anti-inflammatory activities. Even so, organic compounds and active agents in the fruits need to be identified for the plant to be used as herbal drug while the toxicity of the active components, serum-attainable levels, pharmacokinetic attributes, their side effects, and diffusion in various sites around the body also need to be determined. *Nitraria schoberi* fruits decreased and inhibited the growth of food pathogens. Therefore, use of this plant might decrease food poisoning and increase food shelf life.

## Abbreviations

BHA: Butylated hydroxyanisole
DMSO: Dimethyl sulfoxide
MIC: Minimal inhibitory concentration
